# Epstein-Barr Virus_Encoded LMP1 Upregulates MicroRNA-21 to Promote the Resistance of Nasopharyngeal Carcinoma Cells to Cisplatin-Induced Apoptosis by Suppressing PDCD4 and Fas-L 

**DOI:** 10.1371/journal.pone.0078355

**Published:** 2013-10-23

**Authors:** Guang-Da Yang, Tie-Jun Huang, Li-Xia Peng, Chang-Fu Yang, Ran-Yi Liu, Hong-Bing Huang, Qiao-Qiao Chu, Hong-Jie Yang, Jia-Ling Huang, Zhen-Yu Zhu, Chao-Nan Qian, Bi-Jun Huang

**Affiliations:** 1 Department of Experimental Research, State Key Laboratory of Oncology in South China, Cancer Center, Sun Yat-Sen University, Guangzhou, China; 2 Department of Nasopharyngeal Carcinoma, Cancer Center, Sun Yat-Sen University, Guangzhou, China; 3 Department of Pharmacy, Cancer Center, Sun Yat-Sen University, Guangzhou, China; 4 Zhongshan School of Medicine, Sun Yat-Sen University, Guangzhou, China; 5 Department of Nuclear Medicine, the Second People’s Hospital of Shenzhen, Shenzhen, China; 6 School of Pharmaceutical Sciences, Sun Yat-Sen University, Guangzhou, China; 7 Division of Infectious Diseases, Department of Medicine, University of Pennsylvania School of Medicine, Philadelphia, Pennsylvania, United States of America; Cleveland Clinic Foundation, United States of America

## Abstract

Approximately 30% of patients with Epstein-Barr virus (EBV)-positive advanced nasopharyngeal carcinoma (NPC) display chemoresistance to cisplatin-based regimens, but the underlying mechanisms are unclear. The Epstein-Barr virus (EBV)-encoded latent membrane protein 1 (LMP1), a functional homologue of the tumor necrosis factor receptor family, contributes substantially to the oncogenic potential of EBV through the activation of multiple signaling pathways, and it is closely associated with a poorer prognosis for NPC. Recent studies show that EBV infection can induce the expression of many cellular miRNAs, including microRNA-21, a biomarker for chemoresistance. However, neither a link between LMP1 expression and miR-21 upregulation nor their cross talk in affecting chemoresistance to cisplatin have been reported. Here, we observed that stable LMP1-transformed NPC cells were less sensitive to cisplatin treatment based on their proliferation, colony formation, the IC_50_ value of cisplatin and the apoptosis index. Higher levels of miR-21 were found in EBV-carrying and LMP1-positive cell lines, suggesting that LMP1 may be linked to miR-21 upregulation. These data were confirmed by our results that exogenous LMP1 increased miR-21 in both transiently and stably LMP1-transfected cells, and the knock down of miR-21 substantially reversed the resistance of the NPC cells to cisplatin treatment. Moreover, the proapoptotic factors programmed cell death 4 (PDCD4) and Fas ligand (Fas-L), which were negatively regulated by miR-21, were found to play an important role in the program of LMP1-dependent cisplatin resistance. Finally, we demonstrated that LMP1 induced miR-21 expression primarily by modulating the PI3K/AKT/FOXO3a signaling pathway. Taken together, we revealed for the first time that viral LMP1 triggers the PI3K/Akt/FOXO3a pathway to induce human miR-21 expression, which subsequently decreases the expression of PDCD4 and Fas-L, and results in chemoresistance in NPC cells.

## Introduction

Nasopharyngeal carcinoma (NPC), which is prevalent in Southeast China and Southeast Asia, is closely associated with Epstein-Barr virus (EBV) infection, primarily due to the LMP1 oncogene of EBV. NPC is sensitive to radiotherapy and chemotherapy, and can be cured at a rate as approximately 70% [1,2]. However, approximately 30% of the patients will develop distant metastases, and the prognosis for these patients is very poor [3]. The metastatic NPCs usually develop resistance after 6 cycles of cisplatin-based chemotherapy [4]. Little is known about the molecular mechanism behind this resistance. The copy number of EBV DNA is reported to be elevated in patients with metastatic NPC, indicating the revival or more active proliferation of the virus [5,6]. However, it is unclear whether the activity of EBV in NPC cells is responsible for the resistance of the cancer cells to cisplatin-based chemotherapy. 

 EBV, a human herpesvirus, is implicated in a variety of human malignancies, especially NPC, of which nearly 100% of cancerous tissues are EBV positive [7]. In EBV-associated cancers, the EBV infection is predominantly latent. Resistance to apoptosis and immortalization are necessary for EBV to establish its persistent latency in infected host cells [8], which subsequently leads to EBV-related pathogenesis and further tumorigenesis [9]. Thus, some proapoptotic genes, such as p53 [10], PUMA [11] and Fas-L [12], commonly become EBV-regulated targets. Many anti-cancer drugs, including cisplatin and fluorouracil, kill cancer cells through apoptosis-mediated cytotoxic effects and an abundance of apoptosis-related genes are closely associated with chemosensitivity [13,14]. Therefore, some apoptosis-related genes may act as common modulators of the maintenance of viral latency and of chemoresistance in EBV-carrying cells. 

NPC has been shown to exhibit a type II infection latency, and the LMP1 gene is well_defined as an important oncogene of EBV and a poor prognostic biomarker in NPC patients [15,16]. LMP1 acts as a constitutively active receptor mimic of the tumor necrosis factor (TNF) receptor superfamily to stimulate multiple signaling pathways in a ligand-independent manner, including the NFκB, JAK/STAT, p38/MAPK, PI3K/Akt, and ERK-MAPK/JNK pathways [9,17]. Additionally, through the hijacking of various signaling pathways, LMP1 is able to achieve its pleiotropic effects on cell migration [18], proliferation, apoptosis and stemness [19]. 

In addition to coding genes regulated by EBV LMP1 in host cells, recent studies have also identified LMP1 as a modulator of cellular non-coding miRNAs, including miR-29b [20], miR-146a [21], miR-155 [22] and miR-203 [23], expression in both EBV-carrying epithelial cells and B cells. Recent studies indicate that a number of deregulated miRNAs play an important role in cancer initiation and development at the post-transcriptional level [24]. One report found that EBV_encoded EBNA2 may contribute to EBV-induced B-cell transformation, in particular by increasing miR-21 [25]. However, EBV infection is predominantly latent in EBV-associated cancers. In type II latency, exempliﬁed by Hodgkin’s disease and NPC, EBNA1, EBERs, latent membrane proteins_1 and _2A (LMP1 and LMP2A) and the BART microRNAs without EBNA2 are expressed [26]. However, no direct evidence has thus far shown that EBV LMP1 is able to regulate cellular miR-21, a key anti-apoptotic gene in many types of cancers.

 Among the known oncomiRNAs, miR-21 was the only miRNA upregulated in all of the six cancers studied in one report, including breast, pancreas, colon, lung, prostate and stomach cancer [14]. miR-21 mediates anti-apoptotic and metastatic effects by regulating programmed cell death 4 (PDCD4) in colorectal cancer [27], and miR-21 is a downstream effector of AKT that exerts its anti-apoptotic effects via the suppression of Fas ligand in hypoxic conditions [28]. Recent data suggest that PDCD4 may be involved in mitochondrial-mediated apoptosis [29], whereas Fas/Fas-L axis participates in membrane-mediated apoptosis [12]. miR-21 has been identified as a biomarker for chemoresistance in many cancers and may serve as a novel molecular target for cancer chemotherapy [30]. However, miR-21 as a new target for chemotherapy in NPC has not been thoroughly studied. 

 The activation of the PI3K/AKT pathway is very important in tumorigenesis [31] and partly accounts for the oncogenic properties of LMP1 [32,33]. FOXO3a, a downstream transcription factor of the PI3K/AKT pathway, functions as a tumor suppressor gene to repress some oncogenes, such as miR-21 [34]. Moreover, proapoptotic PDCD4 and Fas-L are expressed at lower levels in NPC [35,36], but the reason for their down-regulation in NPC is not understood. Herein, we hypothesize that LMP1-upregulated miR-21 represses PDCD4 and Fas-L in EBV-positive NPC cells by activating the PI3K/AKT/FOXO3a pathway. To test this hypothesis, we evaluated the impact of manipulating LMP1 on the expression of miR-21 in vitro. Our data demonstrate for the first time that LMP1, at least partly by activating the PI3K/AKT/FOXO3a signaling pathway, can induce the expression of miR-21, which in turn targets and reduces the expression of PDCD4 and Fas-L, and thereby confers resistance to cisplatin treatment in NPC cells. Our findings shed novel insights into the regulation of EBV-encoded LMP1 in the chemoresistance of NPC, and implicate potential drug molecular targets for sensitizing NPC cells to cisplatin-induced apoptosis. 

## Results

### 1. LMP1 mediates the upregulation of human anti-apoptotic miR-21

**Figure 1:**
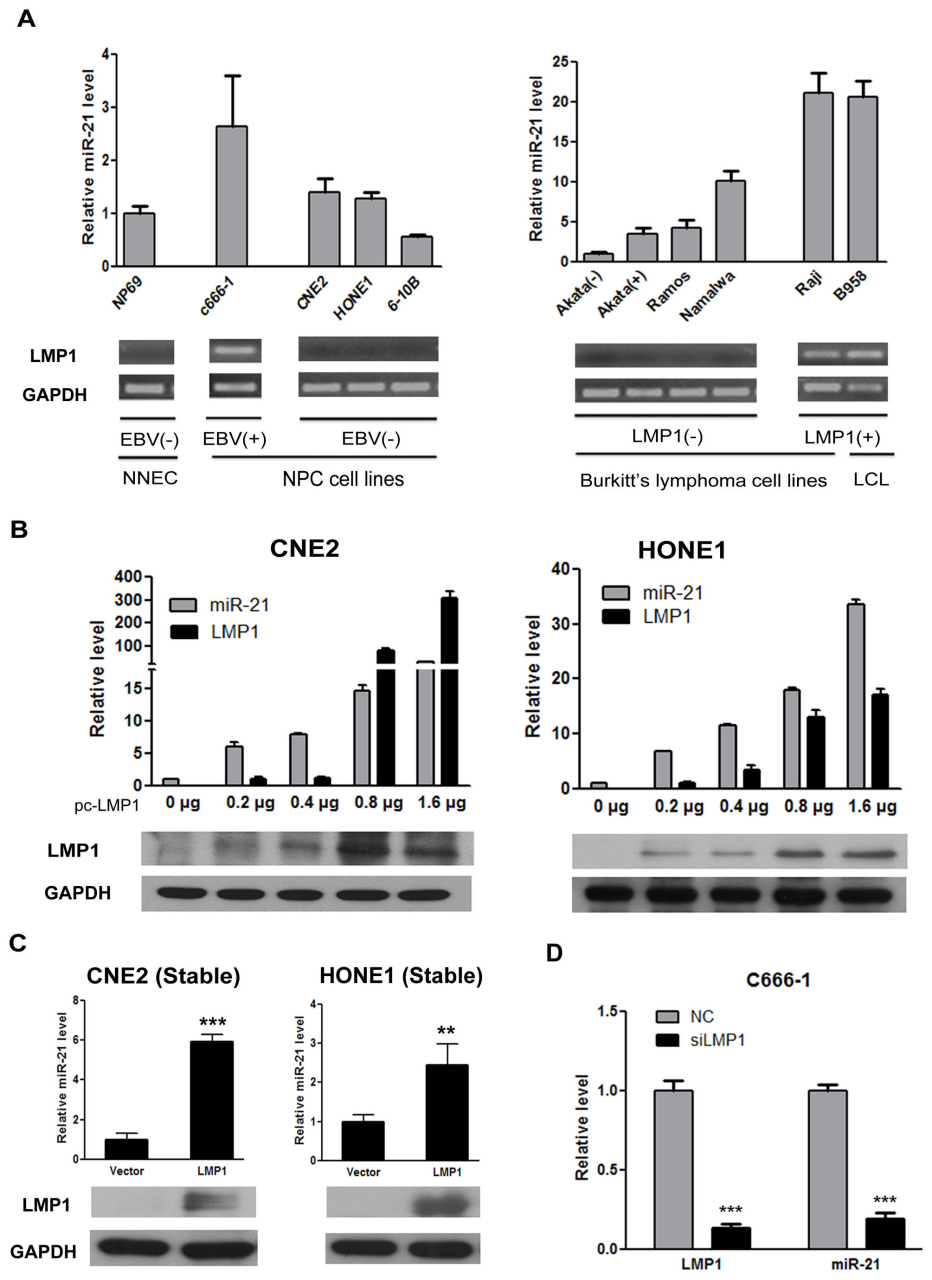
LMP1 promotes the expression of cellular miR-21. (**A**) The relative level of miR-21 transcripts normalized to U6 was determined by quantitative real_time PCR. The data shown are the means ± standard deviation from three independent experiments. The level of miR-21 in NP69 or Akata(-) cells was set as 1. LMP1 expression was detected by RT-PCR in a subset of cancer cell lines with GAPDH as a loading control. (**B**) The LMP1 overexpression plasmid pcDNA3.1-LMP1 (pc-LMP1) was transiently transfected into CNE2 and HONE1 cells. After 48 h, one portion of cells was collected for the analysis of miR-21 and LMP1 expression by qRT-PCR. After 72 h, the remaining cells were harvested for immunoblotting. The successful transfection of the LMP1 overexpression plasmid was also confirmed by immunoblotting for the LMP1 protein. (**C**) The expression of miR-21 was detected in CNE2 and HONE1 stable cell lines. The expression of LMP1 was confirmed by immunoblotting (** *P* < 0.01; *** *P* < 0.001 vs vector control cells). (**D**) C666-1 cells were transfected with siLMP1 or control siRNA (NC) for 48h.The cells was harvested and analyzed for LMP1 and miR-21 expression by qRT-PCR, and the expression levels in control siRNA-transfected cells were set to 1 (*** *P* < 0.001 vs control siRNA- transfected cells). LMP1, latent membrane protein; NNEC, normal NP cell line; LCL, lymphoblastoid cell line.

 To explore whether LMP1 has an effect on miR-21, we first detected the level of miR-21 in NPC and lymphoblastoid cell lines. As shown in the left panels of Figure 1A, EBV_positive C666-1 cells [37] had greater expression of miR-21 than EBV_negative cell lines (the normal NP cell line NP69 [38], CNE2 [39], HONE1 [40] and 6-10B [41]). Similarly, in Burkitt’s lymphoblastoid cell lines or LCL, the increased expression of miR-21 was observed in the cells with endogenous LMP1 expression (B95-8 and Raji); whereas cell lines without detectable levels of LMP1 (Namalwa, Akata (+)[42] and Akata (-)) had lower levels of miR-21 (Figure 1A right panels). These initial experiments suggest that the level of miR-21 may be related to the level of LMP1.

 We then examined miR-21 expression after the transient transfection of the LMP1 plasmid into NPC cells. qRT-PCR and immunoblotting demonstrated that the transient transfection of LMP1 into CNE2 or HONE1 cells increased LMP1 expression at the mRNA and protein levels. LMP1 dose-dependently induced miR-21 expression in the transient transfection experiments (Figure 1B). 

 To validate the observation that LMP1 mediated the upregulation of miR-21, we transfected CNE2 and HONE1 cells with pcDNA3.1-LMP1 or pcDNA3.1 plasmids and selected cells with G418 to establish stable LMP1-expressing cell lines. qRT-PCR analysis showed that LMP1 mRNA was expressed in both of the LMP1-transfected cell lines (Figure S1). The expression of LMP1 protein was detectable by immunoblotting (Figure 1C). In contrast to LMP1-transfected cells, there was no detectable corresponding protein (Figure 1C) in the vector control cells. We further analyzed the levels of miR-21 in stable cell lines and found that expression of miR-21 was approximately six-fold higher in CNE2/LMP1 cells and three-fold higher in HONE1/LMP1 cells compared to vector control cells (Figure 1C). More importantly, we knocked down the endogenous expression of LMP1 in C666-1 cells and found that the expression of miR-21 was also reduced (Figure 1D). These results indicate that LMP1 expression correlates with increased miR-21 levels.

### 2. LMP1 confers resistance to cisplatin-induced apoptosis

**Figure 2:**
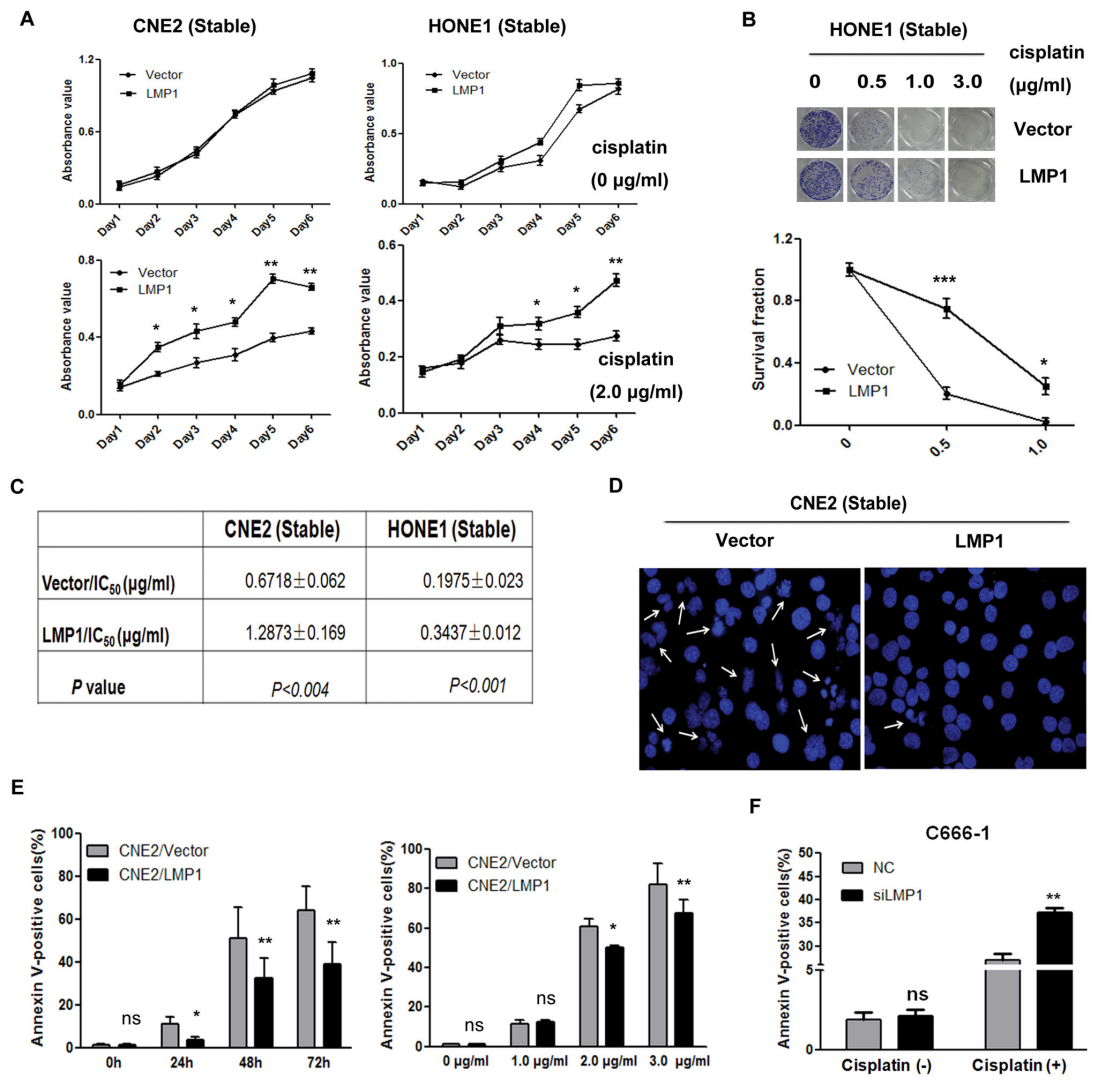
LMP1 confers resistance to cisplatin in NPC cells. (**A**) CNE2 and HONE1 stable cell lines were treated with 0 or 2.0 μg/ml cisplatin. The MTT assay was used to determine the cell proliferation every 24 h for six days (* *P* < 0.05; ** *P* < 0.01 vs vector control cells). (**B**) Colony formation assays were performed for HONE1/LMP1 and HONE1/Vector cells, which were treated with the indicated doses of cisplatin. The upper panel shows the images of the colonies stained by crystal violet. The lower panel is the survival fraction from three independent experiments (* *P* < 0.05, *** *P* < 0.001 vs vector control cells). (**C**) Stably transfected cell lines were treated with various concentrations of cisplatin for 72 h to determine the IC_50_ of cisplatin. The IC_50_ values are presented as the means ± SD of three independent experiments. The IC_50_ is the concentration of chemotherapeutic drug (e.g., cisplatin) that causes a 50% inhibition of tumor cell growth. (**D**) The analysis of apoptosis by Hoechst-33342 staining for disintegrated nuclei in CNE2/LMP1 and CNE2/Vector cells treated with 3 μg/ml cisplatin for 72 h. Representative images (200x) at the indicated time points are shown. The white arrows indicate the cells with obviously broken nuclei. (**E**) The flow cytometry analysis of apoptosis by Annexin V/PI staining in CNE2/LMP1 and CNE2/Vector cells treated with 3 μg/ml cisplatin for indicated time points (left panel) or with cisplatin at various concentrations for 72h (right panel). The histograms shown are the means ± SD from three independent experiments (ns, not significant; * *P* < 0.05; ** *P* < 0.01 vs vector control cells). (**F**) C666-1 cells were transfected with siLMP1 or control siRNA (NC) for 24 h, exposed to 4 μg/ml cisplatin for 48 h or not and assayed to detect the apoptosis rate. The histograms shown are the means ± SD of three independent experiments (ns, not significant; ** *P* < 0.01 vs control siRNA transfected cells).

 The MTT assay was used to assess the effects of LMP1 on the proliferation of NPC cells in the presence of cisplatin. Control vector cells and cells expressing LMP1 showed identical growth curves without cisplatin treatment (Figure 2A). However, 48 hours after plating, the LMP1-transfectants had a significantly accelerated growth rate (up to 144 h) compared to the control vector-transfected cells when the cells were treated with 2.0 μg/ml cisplatin (Figure 2A). Similar results were found in the colony formation assay, as shown in the survival curve of HONE1/LMP1 cells in a dose-escalated cisplatin treatment (Figure 2B). In addition, we determined the IC_50_ of cisplatin and found that LMP1-expressing cell lines were more resistant to cisplatin than the control cells, with an IC_50_ of 1.2873 ± 0.169 μg for CNE2/LMP1 versus 0.6718 ± 0.062 μg for CNE2/Vector (*P* < 0.004) and 0.3437 ± 0.012 μg for HONE1/LMP1 versus 0.1975 ± 0.023 μg for HONE1/Vector cells (*P* < 0.001) (Figure 2C). 

 To clearly define the role of LMP1 in cell apoptosis induced by cisplatin, CNE2/LMP1 and CNE2/Vector cells were treated with cisplatin, and disintegrated nuclei, identified in the staining analysis, were used as an indicator of apoptosis. We found that CNE2/Vector cells have significantly more cells with disintegrated nuclei compared to CNE2/LMP1 cells (Figure 2D). However, without cisplatin treatment, there was no difference between CNE2/LMP1 and CNE2/Vector in their fraction of Annexin V-positive cells. The number of cells that underwent apoptosis after cisplatin treatment was significantly lower in CNE2/LMP1 cells (*P < 0.05 at 24 h, P* < 0.01 at 48 h or 72 h). When we treated the cells with cisplatin at doses up to 3 μg/ml for 72 h, increased proportion of cells underwent apoptosis was observed in a dose_dependent manner. CNE2/LMP1 cells had a lower proportion of apoptotic cells than CNE2/Vector cells in the presence of 2 μg/ml (*P* < 0.05) or 3 μg/ml (*P* < 0.01) cisplatin (Figure 2E). The knock down of LMP1 in C666-1 cells enhanced cisplatin-induced apoptosis (Figure 2F). Similarly, the knock down of LMP1 in CNE2/LMP1 cells reversed cisplatin resistance (Figure S2). These data highlight the important role of LMP1 in the development of cisplatin resistance in NPC cells.

### 3. miR-21 plays an important role in the resistance of cisplatin induced by LMP1

**Figure 3:**
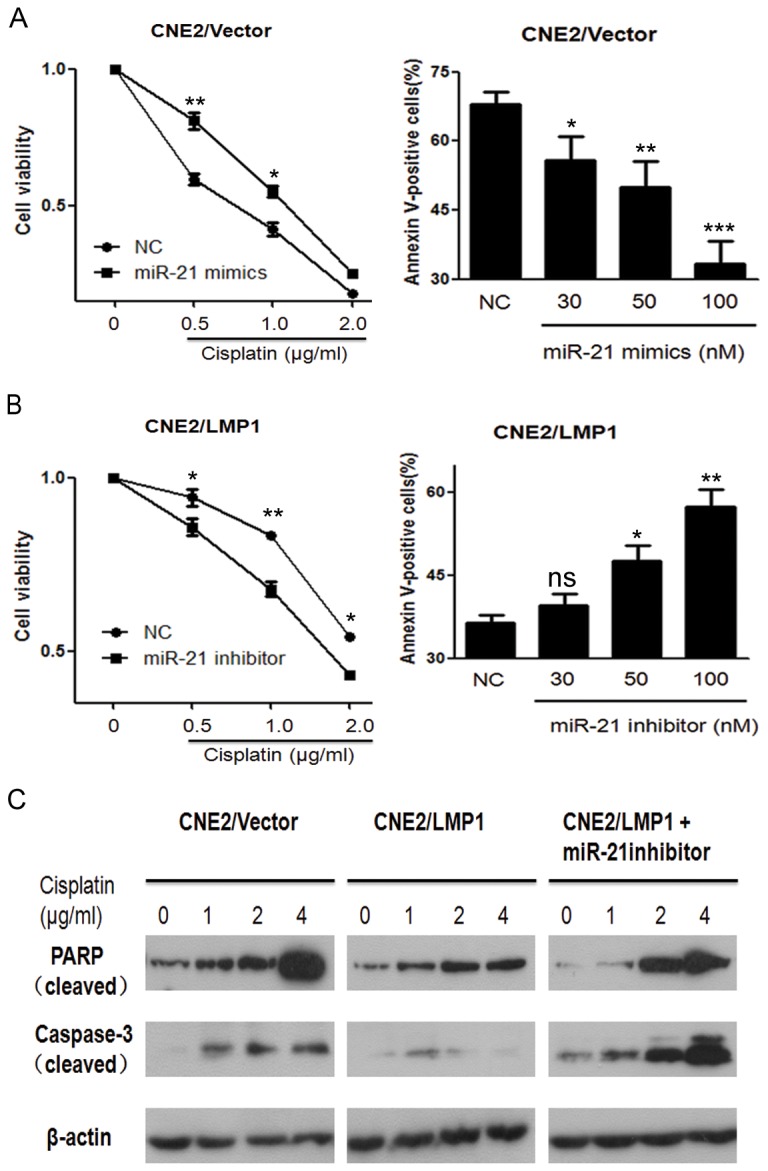
miR-21 plays an important role in cisplatin resistance in NPC cells. (**A**,**B**) CNE2/Vector (A) and CNE2/LMP1 (B) cells transfected with 50 nM miR-21 mimics or inhibitors or negative controls were treated with increasing concentrations of cisplatin for 72h. Cellular viability was analyzed by the MTT assay. CNE2/Vector (A) and CNE2/LMP1 (B) cells were transfected with increasing doses of miR-21 mimics or inhibitors or negative controls for 24 h, and then treated with 3 μg/ml cisplatin for 48 h. Cells were harvested for flow cytometry to detect apoptosis by staining with Annexin V/PI (ns, not significant; * *P* < 0.05; ** *P* < 0.01; *** *P* < 0.001 vs negative control transfected cells). (**C**) CNE2/Vector, CNE2/LMP1 and CNE2/LMP1 cells transfected with miR-21 inhibitor (50 nM for 6h) were treated with increasing doses of cisplatin for 24 h, and then harvested to analyze the expression of cleaved PARP and cleaved caspase-3 by immunoblotting.

 To test the potential function of miR-21 in inducing the resistance of NPC cells to cisplatin treatment, we first increased the expression of miR-21 with specific mimics. The enforced expression of miR-21 reduced sensitivity to the growth inhibition effects of cisplatin, as observed in the cell viability percentage curve at an indicated cisplatin dose (Figure 3A). Moreover, miR-21 dose_dependently enhanced the cellular resistance to cisplatin-induced apoptosis, as evaluated by the Annexin/PI FACS assay (Figure 3A). We then characterized the role of miR-21 in the cisplatin resistance induced by LMP1. CNE2/LMP1 cells transfected with an miR-21 inhibitor were significantly sensitized to cisplatin, as shown in the cell viability curve under the treatment of escalating doses of cisplatin. The knock down of miR-21 contributed to a significant increase of cells undergoing apoptosis in response to cisplatin treatment (Figure 3B). To further investigate the effect of miR-21 on the resistance to cisplatin-induced apoptosis, we studied the levels of both cleaved PARP and cleaved caspase-3 after exposure to cisplatin in CNE2 stable cell lines. As shown in Figure 3C, the cisplatin-treated cells had increased cleaved PARP and cleaved caspase-3 protein levels in a dose-dependent manner. Cells expressing LMP1 had lower levels of cleavage than vector control, confirming that LMP1 confers resistance to cisplatin-induced apoptosis. The inhibition of miR-21 in CNE2/LMP1 cells attenuated the role of LMP1 in cisplatin resistance, as shown by the increase of cleaved PARP and cleaved caspase-3 protein levels to nearly the level observed in cisplatin-treated CNE2/Vector cells. These data indicate that miR-21 plays an important role in LMP1-dependent cisplatin resistance. 

### 4. The effect of miR-21 on cisplatin-induced apoptosis is primarily through the regulation of PDCD4 and Fas-L expression

**Figure 4:**
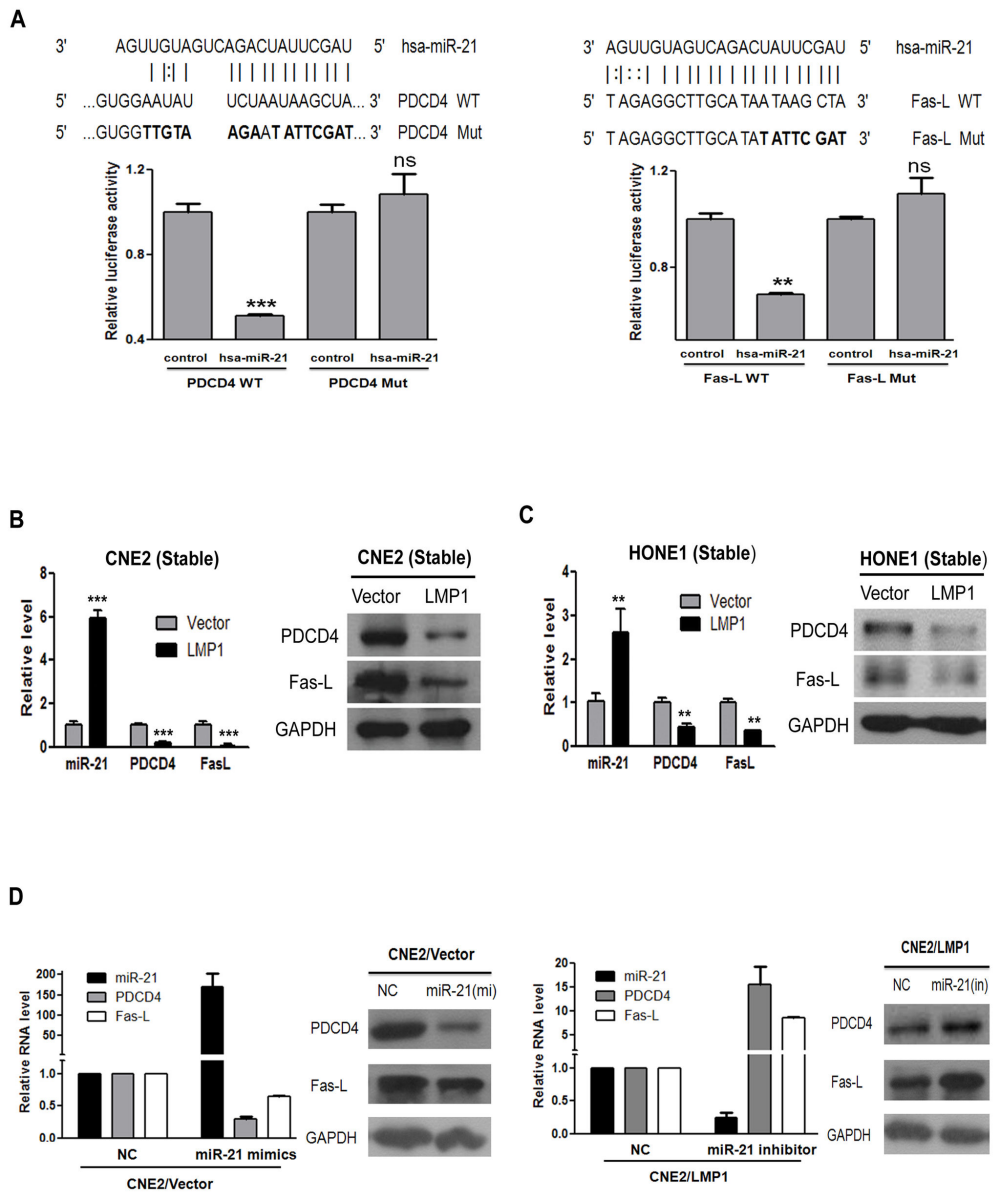
The antiapoptoic effect of miR-21 is through downregulation of PDCD4 and Fas-L. (**A**) Sequence alignment between miR-21 and the 3’UTR of human PDCD4 or Fas-L mRNA. Boldfaced letters indicate the seed-mutated region. Luciferase reporter assays show the effect of miR-21 on the activity of the PDCD4 3’UTR or Fas-L 3’UTR reporters. Co-transfection was performed in 293T cells using plasmids, including human PDCD4 3’UTR (PDCD4 WT), Fas-L 3’UTR (Fas-L WT), the miR-21_binding site-mutated (Mut) PDCD4 3’UTR (PDCD4 Mut), the miR-21_binding site-mutated (Mut) Fas-L 3’UTR (Fas-L Mut), and a miR-21 expression plasmid (pLMP-miR-21). Empty pLMP plasmid was used as a negative control. The values in negative control treated cells were all set to 1 (ns, not significant; ** *P* < 0.01; *** *P* < 0.001 vs negative control treated cells). (**B**, **C**) The levels of miR-21, PDCD4 and Fas-L in CNE2 (B) and HONE1 (C) stable cell lines. The left and right panels show the mRNA and protein levels, respectively. mRNA levels were normalized with GAPDH mRNA levels, and the miRNA level was normalized to that of U6. The values in vector control treated cells were all set to 1 (** *P* < 0.01; *** *P* < 0.001 vs vector control treated cells). (**D**) CNE2 stable cell lines were transfected with 50 nM miR-21 inhibitor (right panels), miR-21 mimics (left panels) or negative controls (NC) and then harvested after 72 h to detect the expression of miR-21, PDCD4 and Fas-L by qRT-PCR and immunoblotting. The fold changes were relative to negative controls, which were set as 1.

 Previous studies indicate that miR-21 plays a role in apoptosis resistance and chemotherapy resistance [43,44], in part through the down-regulation of several proapoptotic genes, including PDCD4 [27] and Fas-L [28]. We thus hypothesized that PDCD4 or Fas-L is a direct miR-21 target. To confirm this hypothesis, the 293T cells were co-transfected with PDCD4-3’UTR or Fas-L-3’UTR luciferase reporters and miR-21. Transfections with empty vector (control) were performed in parallel. As shown in Figure 4A, miR-21 markedly decreased the activity of the PDCD4-3’UTR reporter (*** *P* < 0.001). However, the transfection of the PDCD4-3’UTR mutant did not affect reporter activity (ns, not significant). Similarly, miR-21 decreased the activity of the Fas-L-3’UTR reporter (** *P* < 0.01) but not activity of the Fas-L-3’UTR mutant. Our results also confirmed that miR-21 negatively regulated PDCD4 and Fas-L (Figure S3). However, little is known about the miR-21-specific downstream target(s) and oncogenic event(s) that contributed to LMP1-dependent NPC cell functions. Herein, we analyzed the mRNA level of PDCD4 and Fas-L in both stable cell lines by qRT-PCR. The results showed that LMP1_expressing cells had higher levels of miR-21, but lower levels of PDCD4 and Fas-L. Western blotting also confirmed that the levels of PDCD4 and Fas-L were reduced in LMP1_expressing cells (Figure 4B and 4C). 

 To further verify these findings, we artiﬁcially overexpressed miR-21 via a mimic, or knocked it down with an inhibitor in CNE2 stable cell lines. We found that enforced expression of miR-21 reduced the expression of PDCD4 and FasL at both the mRNA and protein levels. Meanwhile, the suppression of endogenous miR-21 caused the upregulation of endogenous PDCD4 and Fas-L (Figure 4D). The relationship of miR-21 with PDCD4 and Fas-L was also confirmed by the observation that the knock down of LMP1 was accompanied by the downregulation of miR-21 and the upregulation of PDCD4 and Fas-L (Figure S4). Interestingly, both CNE2/LMP1 and CNE2/Vector cells had an increased expression of PDCD4 and Fas-L in response to cisplatin treatment, suggesting PDCD4 and Fas-L are the effectors for cisplatin [45,46] in NPC cells. The knock down of miR-21 in CNE2/LMP1 cells partially reversed PDCD4 and Fas-L expression (Figure S5). Therefore, miR-21 can target PDCD4 and Fas-L, which may be involved in cisplatin resistance induced by LMP1.

### 5. LMP1 induces miR-21 expression primarily by activating the PI3K/AKT/FOXO3a signaling pathway

**Figure 5:**
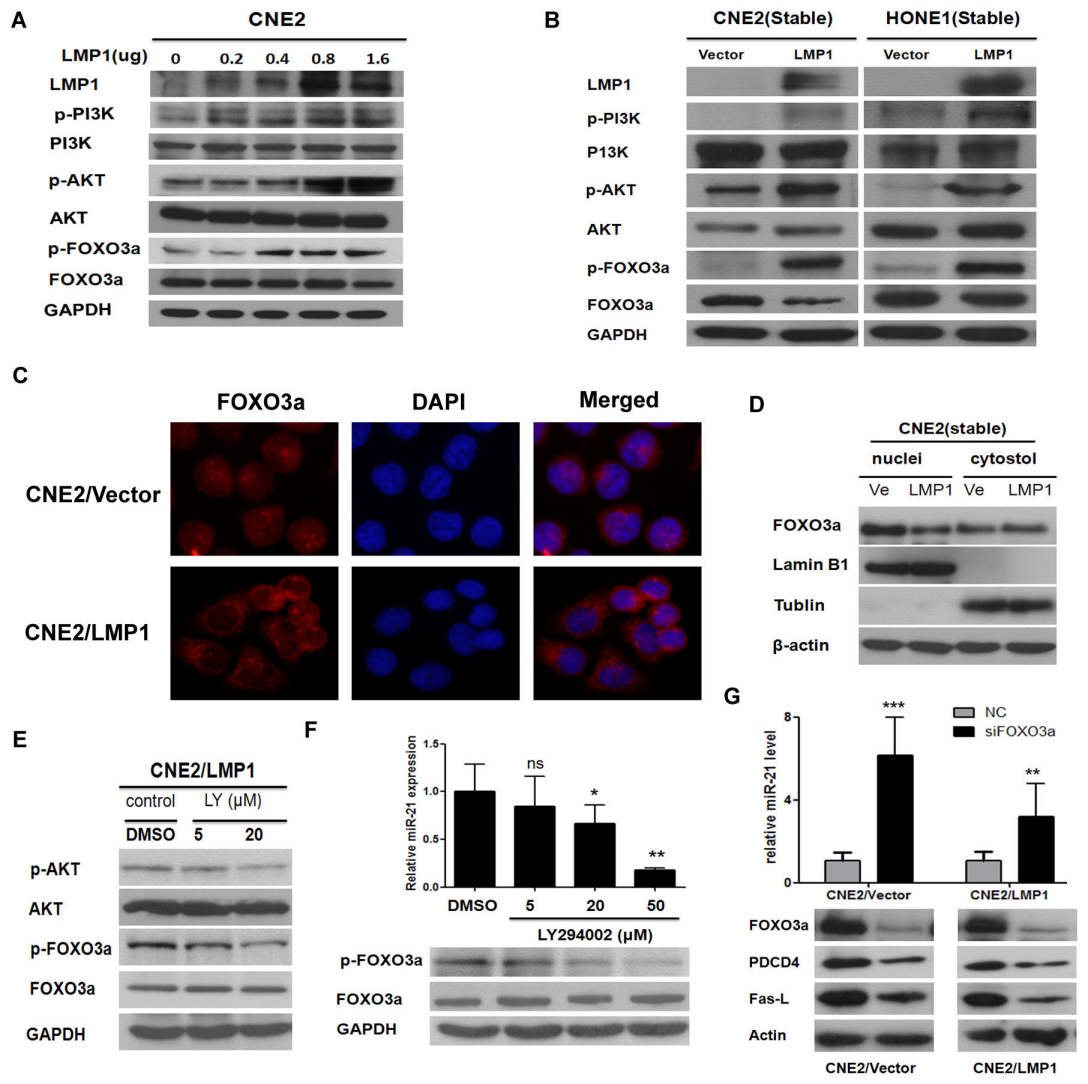
LMP1 expression results in the activation of the PI3K/AKT pathway and the inactivation of FOXO3a. (**A**, **B**) Western blot analysis for LMP1, p-PI3K, PI3K, p-AKT, AKT, p-FOXO3a and FOXO3a expression in cells treated as in Figure 2B and 2C. (**C**) CNE2/Vector, CNE2/LMP1 cells were harvested for the analysis of FOXO3a by immunofluorescent staining. Cell nuclei were stained with 4’, 6-diamidino-2-phenylindole (DAPI). (**D**) CNE2/LMP1 and CNE2/Vector (Ve) cells were collected for the analysis of total FOXO3a by immunoblotting the nuclear and cytosolic fractions. Lamin B1 and Tubulin were used as controls for nuclear and cytoplasmic compartment, respectively, and β-actin was used as a loading control for both fractions. (**E**) CNE2/LMP1 cells were serum_starved for 24 h and treated with LY294002 (LY) at indicated doses for 12 h. pAKT, AKT, pFOXO3a, FOXO3a and GAPDH (loading control) were detected by immunoblotting. (**F**) To analyze the association between p-FOXO3a and miR-21, western blot analyses of p-FOXO3a, FOXO3a and GAPDH, or real-time PCR analysis for miR-21 were performed in CNE2/LMP1 cells treated as in (E). The value of miR-21 in DMSO_treated cells was designated as 1 (ns, not significant, * *P* < 0.05, ** *P* < 0.01 vs DMSO_treated cells). (**G**) Both CNE2/Vector and CNE2/LMP1 cells were transfected with 50nM siFOXO3a or negative control. FOXO3a, PDCD4 and Fas-L were detected by immunoblotting. The level of miR-21 was analyzed by qRT-PCR. The value of miR-21 in negative control treated cells was designated as 1 ( ** *P* < 0.01, *** *P* < 0.001 vs negative control treated cells).

 To explore how LMP1 upregulates miR-21, we focused on the PI3K/AKT pathway, a well-known signaling pathway that can be activated by LMP1. Consistent with previously published findings [32,33], we found that the transient expression of LMP1 in CNE2 cells (Figure 5A) activated PI3K and, Akt in a dose_dependent manner, accompanied by the accumulation of FOXO3a phosphorylation. The stable expression of LMP1 was also accompanied by the increased phosphorylation of PI3K, Akt and FOXO3a (Figure 5B). 

 We further investigated the LMP1-derived effect on FOXO3a localization. The immunofluorescence revealed that FOXO3a was translocated from the nuclei to the cytoplasm in the presence of LMP1 (Figure 5C). The immunoblotting of nuclear and cytoplasmic compartments also confirmed that FOXO3a was markedly distributed in the nuclei in the control cells (Figure 5D). In addition, LY294002 was used to treat LMP1-expressing cells. As shown in Figure 5E, LY294002 abolished the LMP1-induced increase in the levels of phospho-AKT and downstream phospho-FOXO3a. More excitingly, miR-21 expression was also decreased in CNE2/LMP1 cells treated with LY294002 (Figure 5F). As expected, LY294002 partially abolished the effect of LMP1 on the resistance to cisplatin-induced apoptosis (Figure S6).

 A recent report has shown that FOXO3a regulates apoptosis by negatively targeting miR-21 [34]. This led us to investigate whether the inactivation of FOXO3a by LMP1 has an impact on miR-21 expression. We treated CNE2 stable cells with siRNA specific for FOXO3a. We found that the knock down of FOXO3a had resulted in the upregulation of miR-21 (Figure 5G). Taken together, these data suggest that the LMP1-induced inactivation of FOXO3a through the PI3K/AKT signaling pathway ablates FOXO3a transcriptional activity for miR-21 in NPC cells. 

## Discussion

 Platinum-based chemotherapy is the mainstay treatment for metastatic NPC. However, inherent and acquired resistance to cisplatin frequently occurs with an unclear mechanism. The involvement of EBV in this critical issue is suggested by the elevation of serum EBV DNA in patients with distant metastatic NPC [6,47].

 MiRNAs are found to play an important role in tumor formation and progression [24]. MiR-21 is a well-documented oncogene that is elevated in many cancers and contributes to the resistance of malignant tumors to many chemotherapeutic agents, such as paclitaxel, docetaxel, and gemcitabine [30]. Recently, the altered expression and dysfunction of miRNAs have been found in NPC, and EBV infection can affect the expression of cellular miRNAs [48–50]. In accord with a recent study [20], miR-21 has a higher level in LMP1-positive cell lines. We demonstrated that LMP1 can increase miR-21 expression. Expression of miR-21 alone is enough to induce cisplatin resistance in NPC cells, while silencing the oncomiRNA alone is able to attenuate the cisplatin resistance induced by LMP1. Plasma miR-21 can serve as a circulating tumor biomarker for the early diagnosis of NSCLC and is related to the sensitivity to platinum-based chemotherapy [51]. These results suggest that miR-21 upregulation may be an important event in the carcinogenicity of LMP1 and the development of NPC. 

 FOXO3a, a tumor suppressor gene, is a member of the Forkhead O transcription factor (FOXO) family, and it may be a potential therapeutic target candidate [52]. Furthermore, the reduced expression of FOXO3a correlates with poorer survival in patients with NPC [53]. We found that LMP1 can activate the PI3K/AKT pathway and subsequently lead to the phosphorylation of FOXO3a. We observed the translocation of FOXO3a from the nucleus into the cytoplasm in situ by immunofluorescent staining. This translocation was also confirmed by the immunoblotting analysis of nuclear and cytoplasmic compartments. Phosphorylated FOXO3a in the cytoplasm can be degraded [54], which may explain the observation that there are no distinct differences of FOXO3a in the cytoplasm, and the total amount of FOXO3a is reduced in response to LMP1. In addition, knock down of FOXO3a increased miR-21 expression. Combined with the previous findings that FOXO3a negatively regulates miR-21 with regard to initiating apoptosis [34], our data clearly indicate that LMP1 induces miR-21 expression through the inactivation of FOXO3a. 

 Apoptosis plays a critical role in tumorigenesis and is involved in extrinsic receptor and/or intrinsic mitochondrial pathways. The recent data suggest that PDCD4 may be involved in mitochondrially mediated apoptosis [29], whereas the Fas/Fas-L axis takes part in membrane-mediated apoptosis [12]. Cisplatin causes cytotoxicity mainly through its interaction with DNA to form DNA adducts, culminating in the activation of extrinsic and intrinsic apoptosis. Cisplatin can affect membrane fluidity through the activation of the Fas/Fas-L death pathway [55], and the dysregulation of the Fas/Fas-L system may be an important determinant of cisplatin_resistance in ovarian epithelial cancer cells [46]. The PDCD4 effect appears to be specific for cisplatin-induced apoptosis in ovarian cancer cells [45]. In our study, PDCD4 and Fas-L levels were altered in response to cisplatin treatment. In contrast with control cells, LMP1-expressing cells have reduced expression of PDCD4 and Fas-L, independent of cisplatin treatment. miR-21 can partially abolish this effect of LMP1. C666-1 is the only well-known NPC cell line consistently carrying EBV, but the LMP1 protein was not detectable by western blotting, indicating a low level of LMP1 expression [37]. The reason lack of LMP1 detection may be because EBV-encoded BART miRNAs target the 3’ UTR of the LMP1 gene and negatively regulate LMP1 protein expression [56]. Thus, C666-1 may not be the best model for knocking down LMP1; we explored the effect of knocking down LMP1 in LMP1-expressing cells. The fact that LMP1 downregulates PDCD4 and Fas-L through miR-21 not only explains the low levels of PDCD4 and Fas-L observed in NPC [35,36] but also highlights the roles of PDCD4 and Fas-L in LMP1-mediated resistance to cisplatin. Although FOXO3a has been reported to increase or suppress Fas-L expression, one study demonstrated that FOXO3a did not up-regulate Fas-L transcriptionally manner but through the inhibition of miR-21 [34]. The current results that LMP1 inhibits the expression of apoptosis-related genes (PDCD4 and Fas-L) and thereby contributes to cisplatin resistance suggest that assessing PDCD4 and Fas-L level may help predict cisplatin responses in NPC. Previous studies have found that LMP1 have double effects: the cytotoxic and oncogenic effects [57,58]. Although Pu-Yuan Chang et al [59] found that LMP1 can sensitize nasopharyngeal carcinoma cells to genotoxic drugs (cisplatin, etoposide, and adriamycin), but the cell line CG-1 in above study was difference from the cell lines used in our study including the region, histopathology and the way of EBV infection. Guillaume Brocqueville et al [60] found that the cytotoxic and oncogenic effects of LMP1 are not mutually exclusive but may operate sequentially. Thus, further research about this phenomenon needs to be conducted.

**Figure 6:**
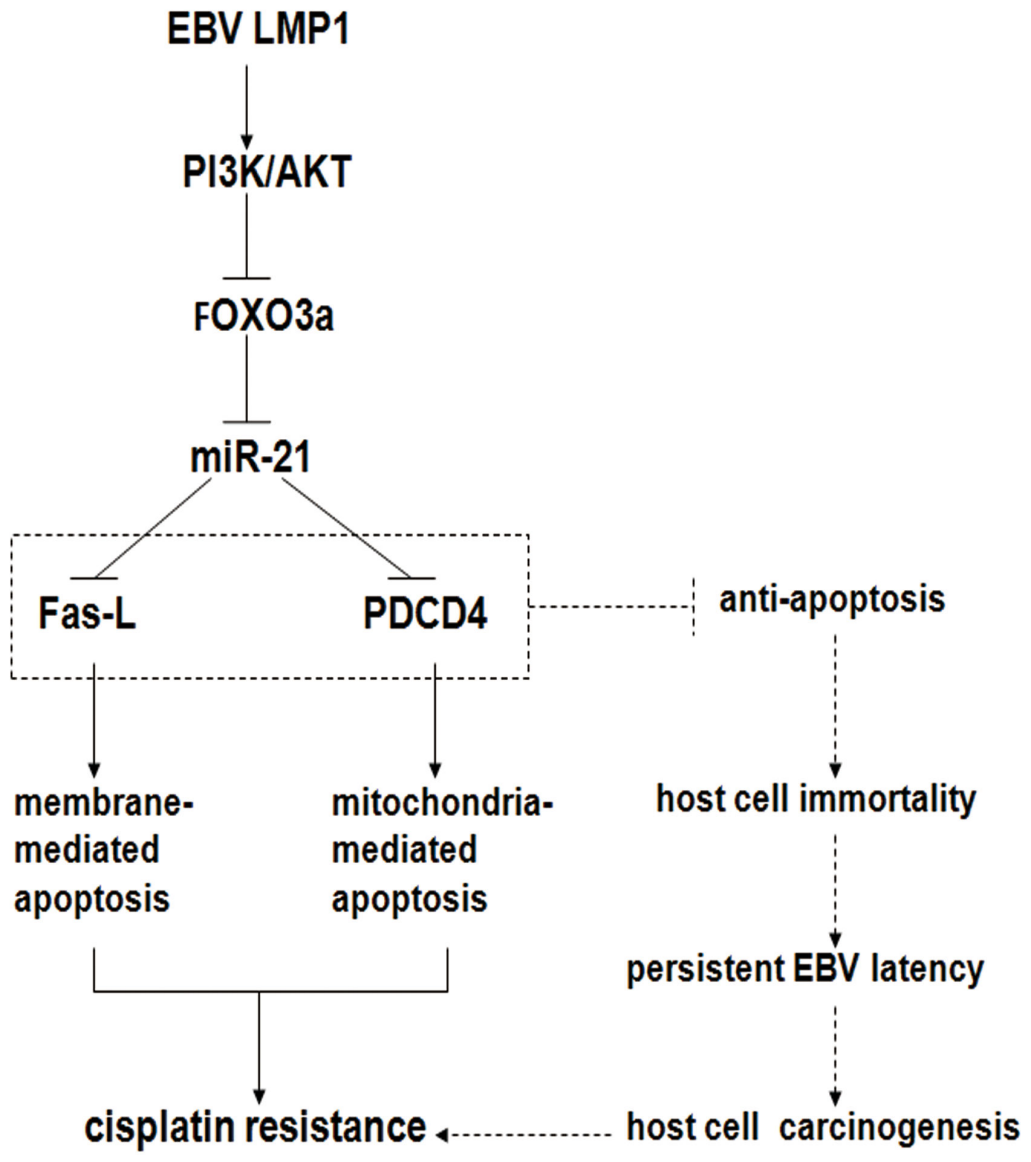
A proposed model for EBV LMP1-mediated miR-21 upregulation in the regulation of specific downstream targets and resulting cisplatin resistance in NPC cells. LMP1 activates the PI3K/AKT signaling pathway, which causes the phosphorylation of FOXO3a. Phosphorylated FOXO3a then translocates from the cytosol to the nucleus and will most likely be degraded. The reduction of FOXO3a in the nucleus results in miR-21 production, which, then functions to reduce PDCD4 and Fas-L expression. These proteins are involved in mitochondrial and membrane-mediated apoptosis, leading to resistance to apoptosis and cisplatin in NPC cells (indicated by the solid lines). The ability to protect host cells from apoptosis is essential for EBV to establish its persistent latent infection, and therefore proapoptotic PDCD4 and Fas-L are usually the key target genes regulated by EBV in latency (indicated by dashed lines). This newly discovered signaling pathway should provide potential drug targets for sensitizing cancer cells to apoptosis and overcoming chemoresistance in LMP1-activated NPC cells.

 EBV actively exploits cellular pathways to regulate the expression of cellular coding and/or non-coding genes to set up a favorable host environment for the virus latency [8]. As summarized in Figure 6, we propose that LMP1 activates the PI3K/AKT signaling pathway, which, in turn, causes the phosphorylation of FOXO3a. Phosphorylated FOXO3a then translocates from the nucleus to the cytosol and loses its transcriptional repression of miR-21. The resultant miR-21 then functions to downregulate PDCD4 and Fas-L, which are involved in mitochondrial [29] and membrane-mediated apoptosis [12], respectively, leading to NPC cell apoptosis resistance, and cisplatin resistance (indicated by solid lines). The ability to protect host cells from apoptosis is essential for EBV to establish its persistent latent infection [8], and therefore PDCD4 and Fas-L are usually the key target genes regulated by EBV in latency (indicated by dashed lines). In conclusion, we not only demonstrate the potential mechanism that accounts for the poor responses of NPC patients to cisplatin at the molecular level, we also further validate the important role of LMP1 in ultimately establishing persistent latent infection for EBV. Nevertheless, further research needs to be conducted because other factors can account for the development of cisplatin resistance.

## Materials and Methods

### Cell lines

 NP69 [38] is a normal nasopharyngeal epithelial cell line, which was transformed with SV40 large T antigen. C666-1, CNE2 [39], HONE1 [40] and 6-10B [41] are nasopharyngeal carcinoma cell lines, of which C666-1 is the only well-known NPC cell line consistently carrying EBV [37], whereas CNE2, HONE1 and 6-10B are EBV_negative. Akata(+), Akata(-), Ramos, Namalwa and Raji are Burkitt ’s lymphoma cell lines, and B95-8 is a lymphoblastoid cell line. B95-8 and Raji are EBV latency III with positive LMP1 expression, while Akata(+) and Namalwa are EBV latency I with negative LMP1 expression [42]. NP69 was grown in keratinocyte SFM medium (Invitrogen, Carlsbad, CA), and the other cells were maintained in RPMI 1640 medium (GIBCO, USA) supplemented with 10% fetal bovine serum and maintained in a humidified atmosphere with 5% CO_2_ at 37°C. 

### Plasmid construction and transformants

 A 3.7-kb fragment containing a full-length LMP1 gene isolated from pZipNeoSV carrying the prototype EBV B95.8 strain LMP1 gene (a gift from Professor Xiao-Feng Zhu, Cancer Center, SYSU, China) using BamHI, and cloned into the BamHI site of pcDNA3.1 to generate pcDNA3.1-LMP1. An empty pcDNA3.1 vector was used as a control.

 For transient transfection, cells were transfected with increasing amounts of pcDNA3.1-LMP1 (0, 0.2, 0.4, 0.8, 1.6 μg/well) using the TurboFectTM in vitro Transfection Reagent (Fermentas) according to the manufacturer’s instructions. Forty-eight or 72 hours after transfection, the cells were harvested for further analysis.

 Both CNE2 and HONE1 cells stably expressing LMP1 were established by transfection with pcDNA3.1 vector or pcDNA3.1-LMP1 construct for 48 h, selected with 500 μg/ml and 300 μg/ml G418 (MERCK, USA), respectively, for 2 weeks, and then maintained in media containing 100 μg/ml G418.

### PI3-kinase inhibitor treatment

 LMP1-expressing cell lines were first serum-starved for 24 h and treated with the PI3-kinase_specific inhibitor LY294002 (Cell Signaling Technology) for another 12 h. The resulting cells were harvested for the analysis of the phosphorylation level of AKT and FOXO3a protein by western blot analysis or miR-21 expression by qRT-PCR. The cells were induced with cisplatin (3 μg/ml) for another 24 h and then were harvested for Annexin V/PI apoptosis assays. FOXO3a expression was also monitored by immunofluorescence staining.

### Oligo RNAs and transfection

 The LMP1, FOXO3a-siRNA and control-siRNAs; miR-21 mimics/inhibitors; and non-speciﬁc scrambled sequences were chemically synthesized in GenePharma Bio-company (ShangHai, China). The sequences are shown in Table S1. The transfections in our study were performed with RNAiMAX Transfection Reagent (Invitrogen) according to the manufacturer’s protocols. 

### Real_time RT-PCR analysis

 Total RNA was isolated with TRIzol reagent (Invitrogen) according to the manufacturer’s instructions. For the analysis of mRNA expression, 1 μg of total RNA was used to produce cDNA using M-MLV reverse transcriptase according to the manufacturer’s instructions (Promega, USA). The resulting cDNAs were amplified using specific primers for LMP1 and GAPDH as an internal control. qRT-PCR was performed using SYBR green (Promega, USA) on Applied Biosystems PRISM 7900HT instruments (Foster City, CA, USA). GAPDH mRNA levels were used for normalization. The All-in-One miRNA qRT-PCR Detection Kit (GeneCopoeia, USA) was used to analyze the expression of non-coding miR-21 and its mimics according to the manufacturer’s protocol. The U6 snRNA was used as an endogenous normalization control. The primers used in our study are shown in Table S2. The relative changes in expression were calculated using the 2^-ΔΔCt^ (where Ct is the threshold cycle) method. 

### Western Blotting

Whole-cell lysates were extracted with RIPA buffer supplemented with 1: 100 protease inhibitors (protease inhibitors cocktail, Sigma-Aldrich). Nuclear or cytoplasmic extracts were prepared with NE-Per (Pierce) in accordance with the manufacturer’s protocol. Primary antibodies specific for PI3K, p-PI3K p85 (Tyr458)/p55 (Tyr199), p-AKT (Ser473), FOXO3a, p-FOXO3a (Ser318/321), cleaved PARP and cleaved caspase-3 were purchased from Cell Signaling, USA. Antibodies specific for AKT, PDCD4, β-actin and Tubulin were purchased from EPITOMICS. Antibodies to LMP1 were obtained from DAKO, and antibody to Fas-L were obtained from Anbo Biotechnology, USA. Antibodies to GAPDH were purchased from Santa Cruz, USA, and Lamin B1 antibodies were from Proteintech, USA. Secondary antibodies used to detect bound proteins include horseradish peroxidase-conjugated anti-mouse and anti-rabbit (Jackson ImmunoResearch Laboratories, USA) antibodies. After treatment with secondary antibodies, blots were developed using the Pierce Super Signal West Pico chemiluminescence system followed by exposure to ﬁlm (KODAK). The protein bands were quantified using Image J 1.33 software (NIH).

### Apoptosis assay

 Apoptosis was analyzed using the Alexa Fluor® 488 annexin V/Dead Cell Apoptosis Kit (Invitrogen) according to the manufacturer’s instructions. Briefly, 1x10^5^ cells treated as indicated in the figure legends were collected and washed twice with PBS. Cells were resuspended in 100 µl 1X annexin-binding buffer. Five microliters of Annexin V and 1 µl of 1X propidium iodide (PI) were added to the cells and then incubated at room temperature for 20 minutes in the dark. Apoptotic cells were then quantified by flow cytometry.

 The Hoechst-33342 staining of disintegrated nuclei was also used for the analysis of apoptosis. Briefly, CNE2/LMP1 and CNE2/Vector cells were treated with 3 μg/ml cisplatin for 72 h and then stained with Hoechst 33342. The stained cells were examined under a ﬂuorescence microscope.

### Colony formation assay

 Stable cell lines were seeded into a 6-well dish at 1000 cells/well and then incubated for 24 h to allow settling. The cells were treated with a range of cisplatin doses (1-3 μg/ml) for 8 h. When most cell colonies had reached >50 cells, they were stained with 0.05% crystal violet. Colonies that consisted of more than 50 cells were scored and compared to the untreated controls. Plating efficiency was determined as the ratio of the number of colonies divided by the number of cells seeded. Each data point represents the mean of three independent experiments.

### MTT assay

 For cell viability assays, the CNE2 stable cell lines were transfected with negative control, miR-21 mimics or miR-21 inhibitor (50 nM) for 24 h. The cells were then plated into 96-well plates at 1000 or 3000 cells/well and treated with cisplatin (0.5-2.0 μg/ml) for 3 days. For cell proliferation assays, stable cell lines seeded into 96-well plates at 1000cells/well were treated with cisplatin (0 or 2.0 μg/ml) for 6 days. For IC_50_ determination, stable cell lines were seeded into 96-well plates at 3000 cells/well and then exposed to cisplatin chemotherapy for 3 days, with cumulative doses ranging from 0 to 25.6 μg/ml. These cells were incubated with 20 μl MTT (5 mg/ml) for 4 h at 37°C. The optical density was measured at 490 nm. The data were analyzed using GraphPad Prism 5 (GraphPad Software, La Jolla, CA, USA). 

### Immunofluorescent Staining

Cells were fixed with 4% paraformaldehyde (Sigma, UK) before being permeabilized in 0.1%Triton X-100. Samples were blocked in PBS containing 5% bovine serum albumin for 60 min and then incubated overnight with the primary rabbit FOXO3a antibody (1:200 Cell Signaling, UK). Following washes with PBS, secondary antibodies conjugated to Alexa 597 (1:500, anti-rabbit, Santa Cruz, USA) were added to the samples for one hour. Cells were counterstained with DAPI (Sigma UK) before mounting. Images were captured using the ﬂuorescence microscope.

### Luciferase assay

 To construct a plasmid expressing miR-21, a fragment containing the miR-21 precursor was cloned into a pLMP vector to create pLMP-miR-21. An empty pLMP vector was used as the negative control (control). A 496 bp fragment of the 3’UTR of human PDCD4 or a 585 bp fragment of the 3’UTR of human Fas-L containing the putative miR-21 binding site was cloned into pLUC (Promega) to create pLUC-PDCD4 WT and pLUC-Fas-L WT, respectively. The site-directed mutagenesis of the miR-21 target-site in PDCD4-3’-UTR or Fas-L-3’-UTR produced pLUC-PDCD4 Mut and pLUC-Fas-L Mut, respectively. For reporter assays, 293T cells were transiently transfected with wild type (WT) or mutant (Mut) reporter plasmid and miR-21 expression plasmids (pLMP-miR-21) or empty vectors (pLMP) using FuGENE® HD (Roche). Luciferase assays for both firefly and renilla luciferase were performed 48 h after transfection with the Dual-Luciferase Reporter Assay System (Promega). The renilla luciferase readings were normalized to the firefly luciferase activity in the corresponding well.

### Statistics

 Student's T-test was used to evaluate the significance between any two groups of data with the statistical package SPSS 17.0 in all the pertinent experiments. A p value <0.05 (using a two-tailed paired t-test) was considered significant.

## Supporting Information

Figure S1 **The mRNA level in C666-1 and LMP1-transfected cells.** The mRNA level in C666-1 and LMP1-transfected cells was analyzed by qRT-PCR, and the value in C666-1 cells was set to 1. Data shown are the means ± SD of three independent experiments (ns, not significant vs C666-1 cells).
(TIF)

Figure S2 **The reversion of cisplatin resistance by knocking down LMP1 in CNE2/LMP1 cells.** CNE2/LMP1 cells were transfected with siLMP1 or control siRNA (NC) for 48 h. The cells were exposed to cisplatin for another 48 h and assayed to detect the apoptosis rate. Data shown are the means ± SD of three independent experiments (** *P* < 0.01 vs control siRNA transfected cells).
(TIF)

Figure S3 **miR-21 negatively regulates the expression of PDCD4 and Fas-L in CNE2 cells.** (**A**, **B**) CNE2 cells were transfected with miR-21 mimics (A) or miR-21 inhibitor (B) or their scrambled control (Scr) and then harvested to detect the expression of miR-21, PDCD4 and Fas-L. The fold changes were relative to the untreated controls (UC), to which a value of 1.0 was assigned.
(TIF)

Figure S4 **The effect of knocking down LMP1 in CNE2/LMP1 cells on the expression of miR-21, PDCD4 and Fas-L.** CNE2/LMP1 cells were transfected with siLMP1 or control siRNA (NC) for 48 h.The cells was then harvested and analyzed for LMP1, miR-21, PDCD4 and Fas-L by qRT-PCR, and the expression levels in control siRNA transfected cells was set to1.
(TIF)

Figure S5 **The expression of PDCD4 and Fas-L in response to cisplatin treatment.** Cells treated as in Figure 3C (but without 0 μg/ml cisplatin treatment) were tested for the expression of PDCD4 and Fas-L by immunoblotting. The protein bands were quantified using Image J 1.33 software (NIH).
(TIF)

Figure S6 **LY294002 can reverse LMP1-induced cisplatin resistance.** (**A**, **B**) CNE2/LMP1 (A) and HONE1/LMP1 (B) cells were treated with DMSO as a control or 5-50 μM LY294002 in the presence of 3 μg/ml cisplatin for 24 h, respectively. The percentage of apoptotic Annexin V-positive cells is presented as bar graphs and the data shown are the means ± SD (ns, not significant; * *P* < 0.05; ** *P* < 0.01; *** *P* < 0.001 vs DMSO treated cells).
(TIF)

Table S1 **The sequence of oligo RNAs used in the study.**
(TIF)

Table S2 **The specific PCR primers used in the study.**
(TIF)
