# Supplementary figures and images for: Epstein-Barr Virus_Encoded LMP1 Upregulates MicroRNA-21 to Promote the Resistance of Nasopharyngeal Carcinoma Cells to Cisplatin-Induced Apoptosis by Suppressing PDCD4 and Fas-L
Source: PLoS One. 2013 Oct 23;8(10):e78355. doi: 10.1371/journal.pone.0078355 (PMC3806812; doi:10.1371/journal.pone.0078355)

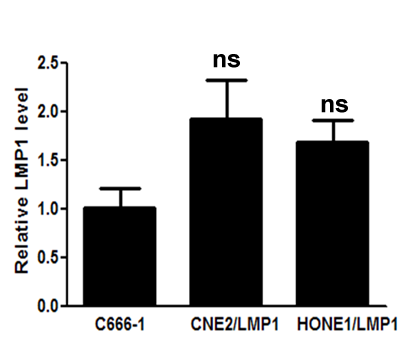

Supplement: Figure S1 — The mRNA level in C666-1 and LMP1-transfected cells. The mRNA level in C666-1 and LMP1-transfected cells was analyzed by qRT-PCR, and the value in C666-1 cells was set to 1. Data shown are the means ± SD of three independent experiments (ns, not significant vs C666-1 cells). (TIF) [file pone.0078355.s001.tif]

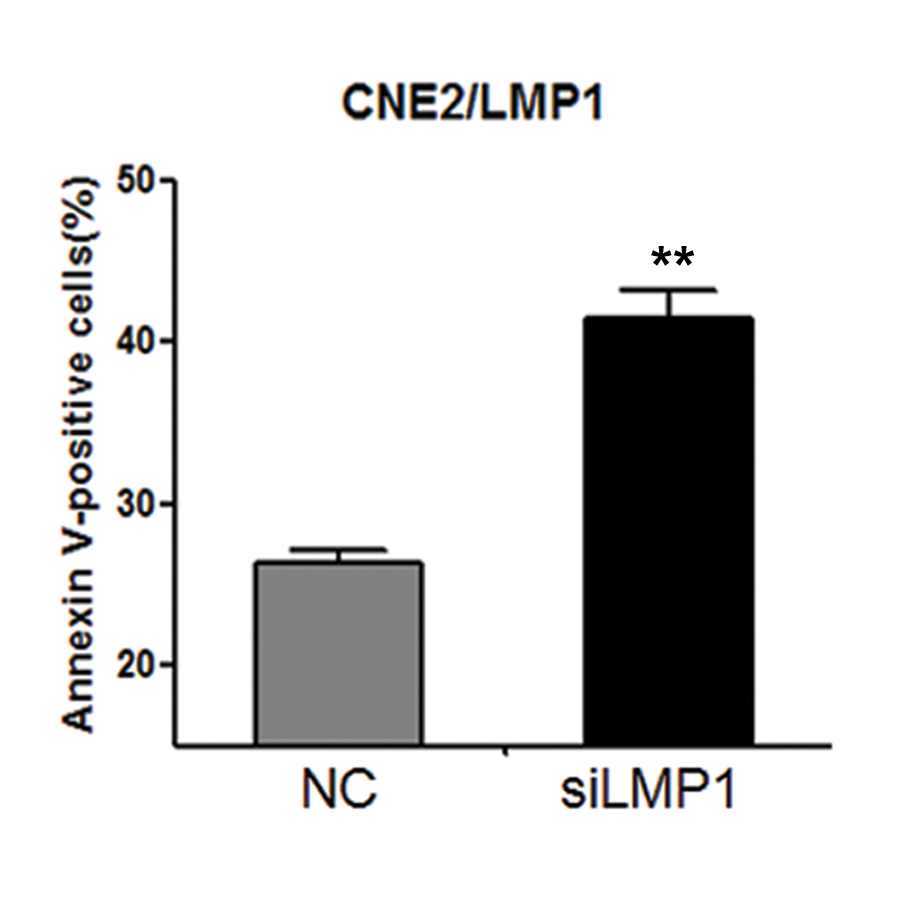

Supplement: Figure S2 — The reversion of cisplatin resistance by knocking down LMP1 in CNE2/LMP1 cells. CNE2/LMP1 cells were transfected with siLMP1 or control siRNA (NC) for 48 h. The cells were exposed to cisplatin for another 48 h and assayed to detect the apoptosis rate. Data shown are the means ± SD of three independent experiments (** P < 0.01 vs control siRNA transfected cells). (TIF) [file pone.0078355.s002.tif]

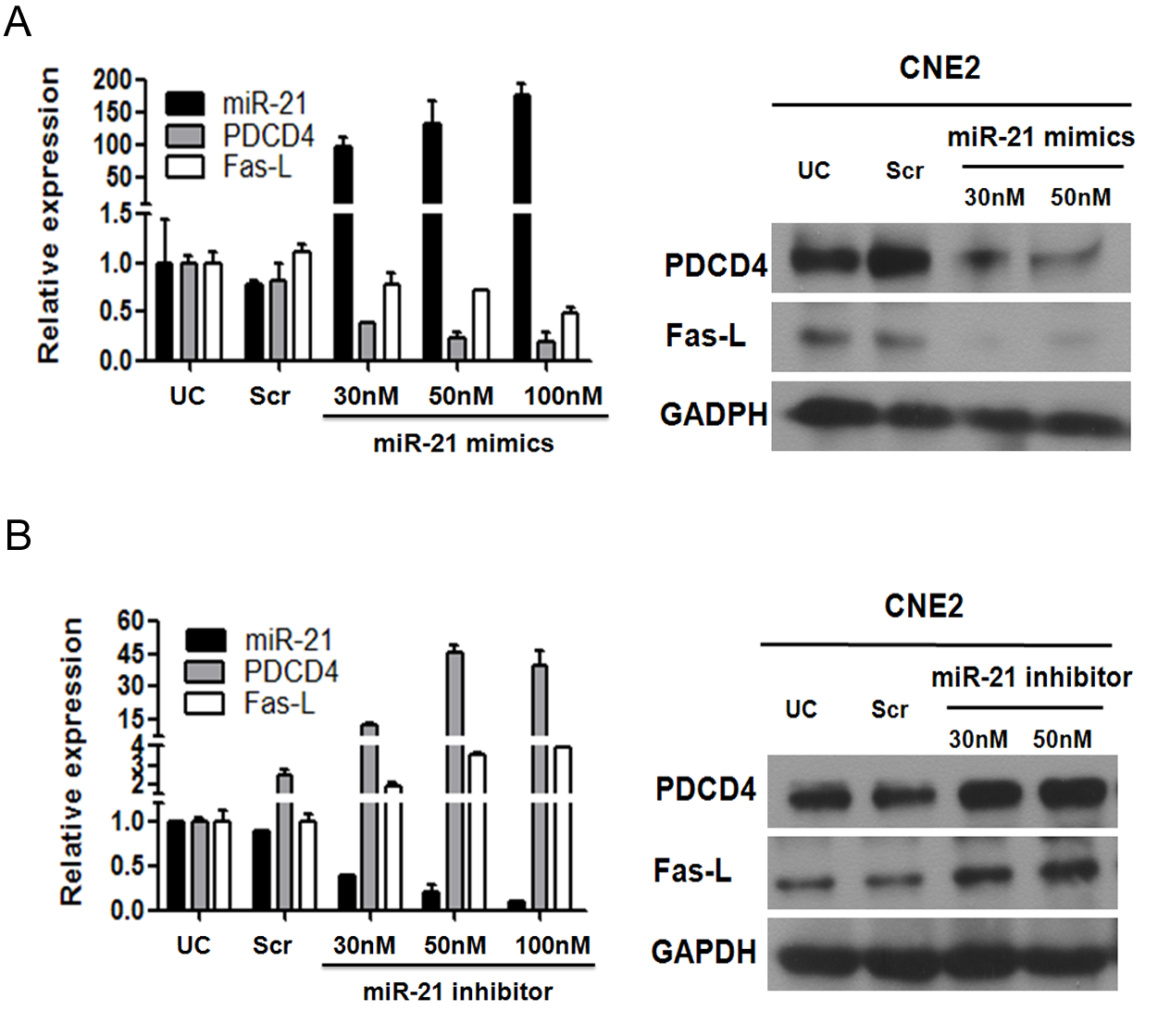

Supplement: Figure S3 — miR-21 negatively regulates the expression of PDCD4 and Fas-L in CNE2 cells. (A, B) CNE2 cells were transfected with miR-21 mimics (A) or miR-21 inhibitor (B) or their scrambled control (Scr) and then harvested to detect the expression of miR-21, PDCD4 and Fas-L. The fold changes were relative to the untreated controls (UC), to which a value of 1.0 was assigned. (TIF) [file pone.0078355.s003.tif]

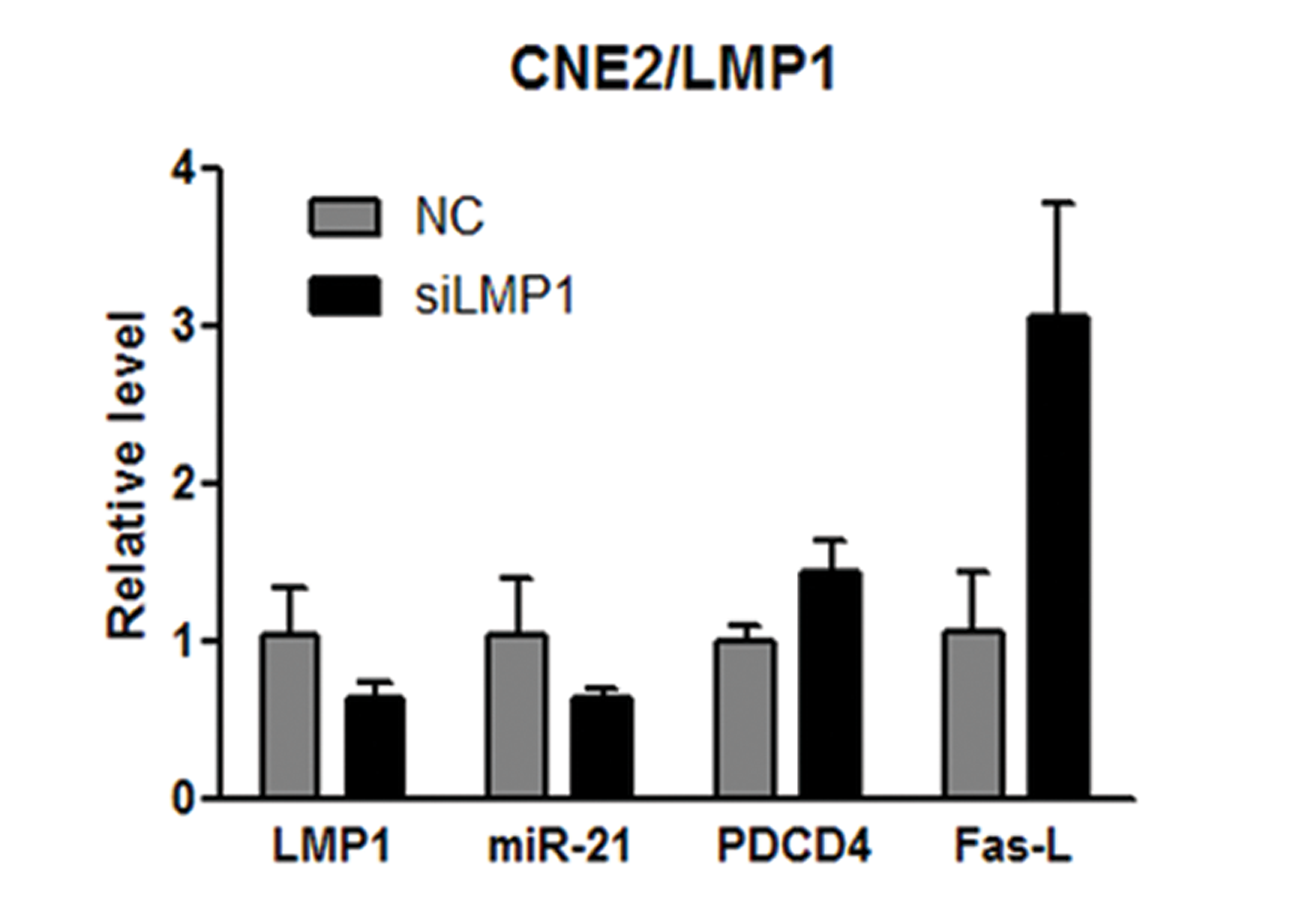

Supplement: Figure S4 — The effect of knocking down LMP1 in CNE2/LMP1 cells on the expression of miR-21, PDCD4 and Fas-L. CNE2/LMP1 cells were transfected with siLMP1 or control siRNA (NC) for 48 h.The cells was then harvested and analyzed for LMP1, miR-21, PDCD4 and Fas-L by qRT-PCR, and the expression levels in control siRNA transfected cells was set to1. (TIF) [file pone.0078355.s004.tif]

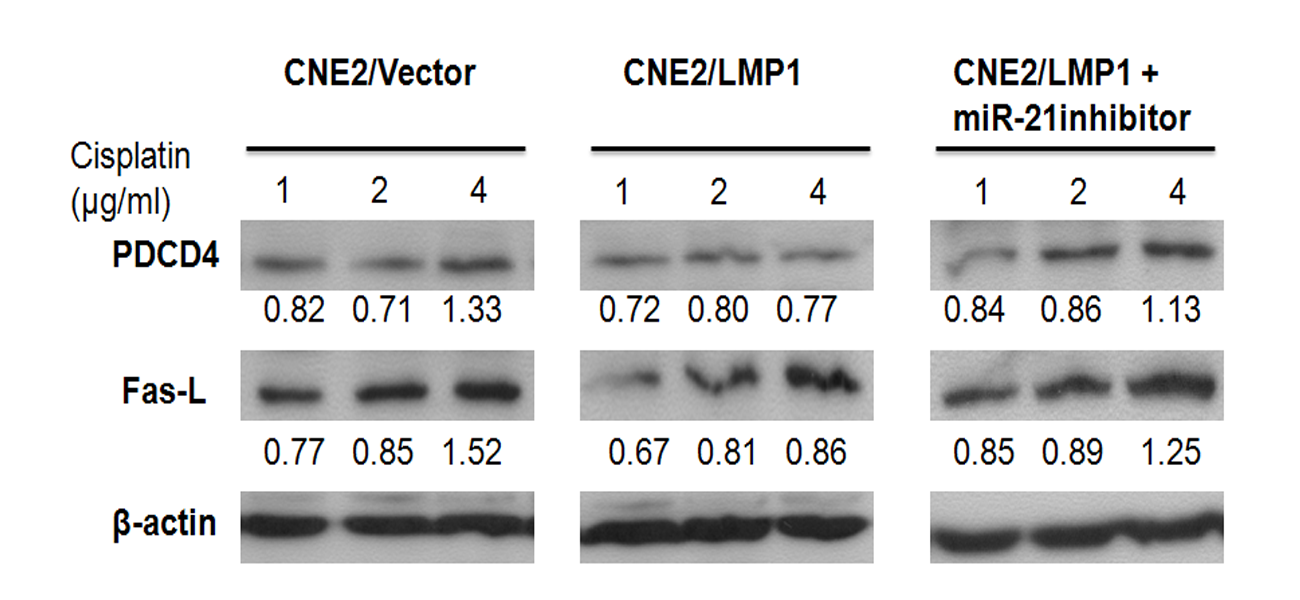

Supplement: Figure S5 — The expression of PDCD4 and Fas-L in response to cisplatin treatment. Cells treated as in Figure 3C (but without 0 μg/ml cisplatin treatment) were tested for the expression of PDCD4 and Fas-L by immunoblotting. The protein bands were quantified using Image J 1.33 software (NIH). (TIF) [file pone.0078355.s005.tif]

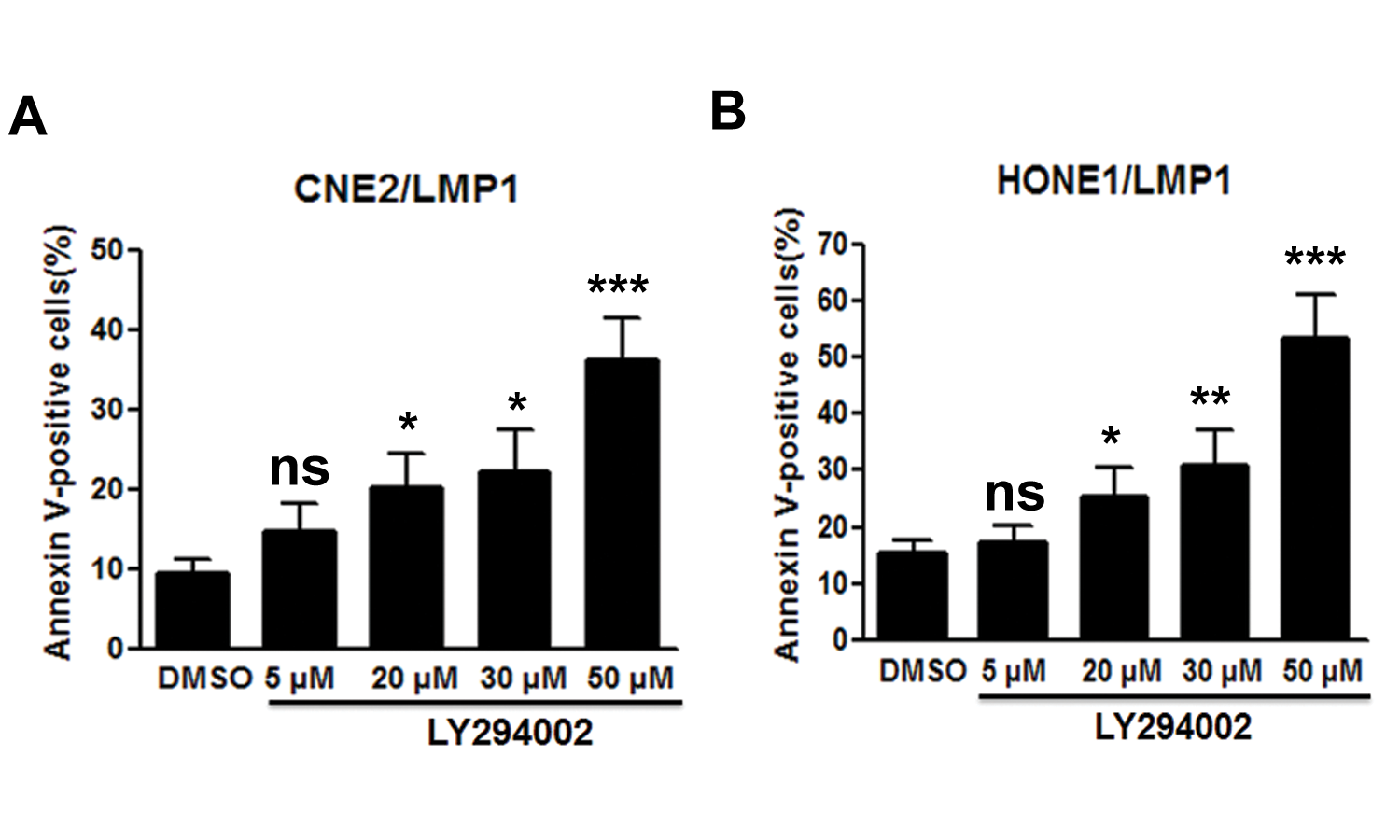

Supplement: Figure S6 — LY294002 can reverse LMP1-induced cisplatin resistance. (A, B) CNE2/LMP1 (A) and HONE1/LMP1 (B) cells were treated with DMSO as a control or 5-50 μM LY294002 in the presence of 3 μg/ml cisplatin for 24 h, respectively. The percentage of apoptotic Annexin V-positive cells is presented as bar graphs and the data shown are the means ± SD (ns, not significant; * P < 0.05; ** P < 0.01; *** P < 0.001 vs DMSO treated cells). (TIF) [file pone.0078355.s006.tif]

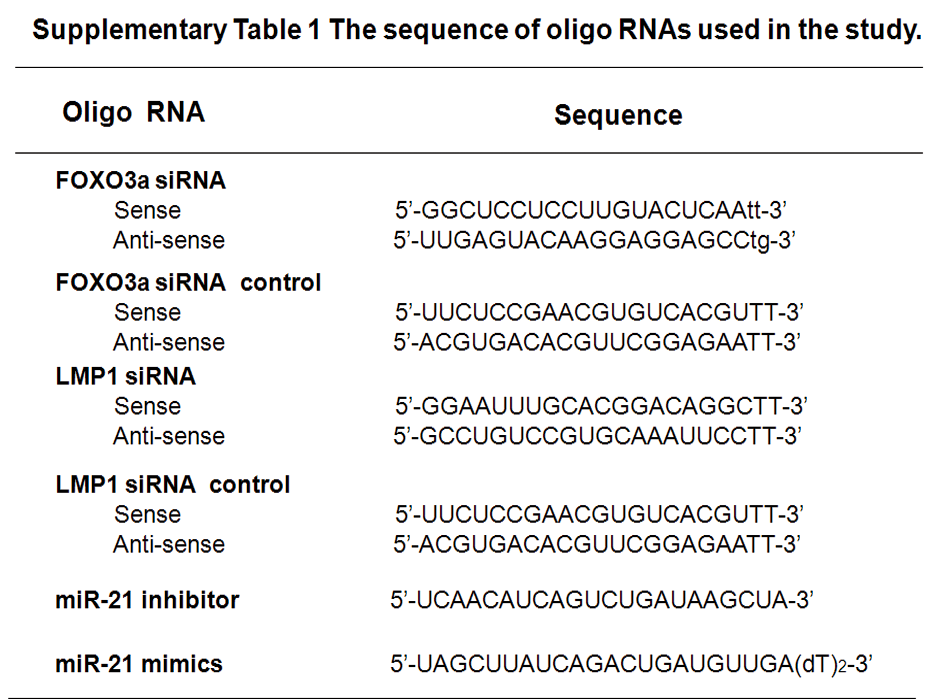

Supplement: Table S1 — The sequence of oligo RNAs used in the study. (TIF) [file pone.0078355.s007.tif]

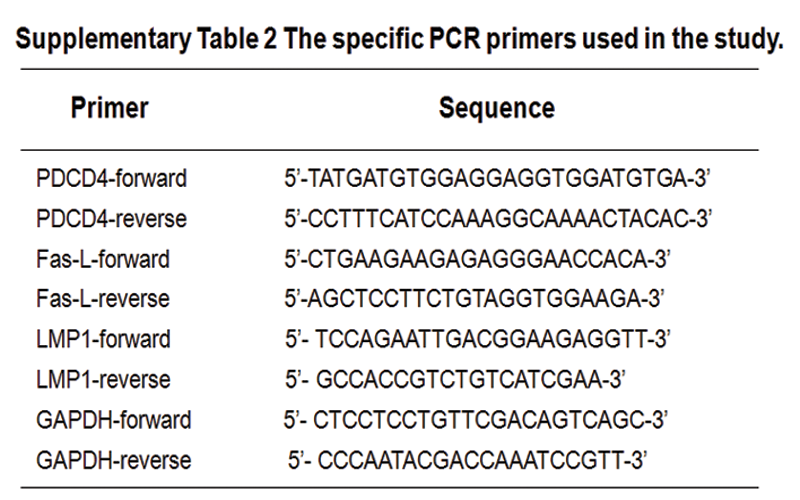

Supplement: Table S2 — The specific PCR primers used in the study. (TIF) [file pone.0078355.s008.tif]
